# Mode I Interlaminar Fracture of Glass/Epoxy Unidirectional Laminates. Part I: Experimental Studies

**DOI:** 10.3390/ma12101607

**Published:** 2019-05-16

**Authors:** Sylwester Samborski, Adrian Gliszczynski, Jakub Rzeczkowski, Nina Wiacek

**Affiliations:** 1 Department of Applied Mechanics, Faculty of Mechanical Engineering, Lublin University of Technology, Nadbystrzycka 36, 20-618 Lublin, Poland; kubarzeczkowski@op.pl; 2 Department of Strength of Materials, Faculty of Mechanical Engineering, Lodz University of Technology, Stefanowskiego 1/15, 90-924 Lodz, Poland; gliszczynski@p.lodz.pl (A.G.); nina.wiacek@gmail.com (N.W.)

**Keywords:** double cantilever beam (DCB), Mode I, interlaminar fracture, GFRP, acoustic emission, strain energy release rate

## Abstract

The paper presents experimental tests of unidirectional double cantilever beams made of a glass fiber reinforced (GFRP) laminate. The critical value of the strain energy release rate (c-SERR or *G*_IC_), i.e., the mode I fracture toughness of the considered material was determined with three different methods: the compliance calibration method (CC), the modified compliance calibration method (MCC), and the corrected beam theory (CBT). Due to the common difficulties in precise definition of delamination initiation force, the Acoustic Emission (AE) technique was applied as an auxiliary source of data. The failure process was monitored, as well, in order to detect and identify different damage phenomena. This was achieved through a detailed analysis of the raw AE signal subjected to fast Fourier transformation (FFT). The frequency spectra revealed three dominating frequency bands with the basic one described by the average value of 63.1 kHz, revealing intensive delamination processes. This way, not only precise values of the critical SERR, but also the information on damage evolution during propagation of delamination, was obtained.

## 1. Introduction

Fracture or delamination along an interface between phases plays a major role in limiting the toughness and the ductility of multi-phase materials, such as fibre-matrix composites and laminated composite structures. Considerable efforts have, therefore, been devoted to the characterization of delamination resistance, with a clear prevalence of fracture mechanics approaches [1,2]. Mode I fractures have received the greatest attention and various standards have been developed for the double cantilever beam (DCB) test [3,4,5]. In opposition to embedded fiber Bragg grating sensors and crack gauges, image processing techniques are becoming widely used for facilitating the post-processing of static and fatigue mode I tests [6]. The mode I interlaminar fracture test is also used to compare the composite bonded joints connected by different epoxy adhesives [7,8,9,10]. Currently, tests in mode I are also conducted in significantly different conditions to those resulting from the application of the standards, e.g., by an increased loading rate [11] or even dynamic fracture tests [12] (with the use of dual electromagnetic Hopkinson bar) have been performed.

In most real applications, transverse matrix cracking and delamination are intrinsically associated and constitute a typical damage mechanism of composites, especially when structures are submitted to bending loads [13,14]. A typical example of this phenomenon is a low velocity impact test. In the vast majority of papers, one can find information that the force needed to initiate delamination is lower than the force needed to initiate matrix cracking [15]. Nevertheless, even if the first element that degrades is the matrix of a particular layer, the transverse crack in the matrix immediately propagates towards the other layers. In most practical applications, laminates are characterized by a multidirectional arrangement of fibers. Thus, if the transverse crack of the matrix reaches adjacent layers, propagation of damage changes direction leading to interlaminar delamination. On the other hand two adjacent laminae having different fibre angles induce an extensional and bending stiffness mismatch which, when combined with the low strength of the matrix, make composite materials very sensitive to the delamination at those interfaces.

Although most contemporary literature focuses on the behavior of delaminating interface in multidirectional laminates [16,17] there are still works devoted to unidirectional laminates. Szekréyes and Uj [18] have investigated the interlaminar fracture and fiber-bridging in double-cantilever unidirectional beam specimens from theoretical and experimental points of view. They have stated that the well-known classical beam theory-based solution agrees excellently with the experimental results in the case of crack initiation tests, while it seems to be inadequate for the evaluation of the propagation test data. Based on the solution of beam theory, they have approximated the number of the bridging fibers and the bridging force, which has allowed determination of a plateau dependence of the energy release rate in the function of the crack length as the crack grows. De Moura et al. [19] demonstrated that the intrinsic toughness without fibre bridging is similar for interlaminar and intralaminar cases. This is an important conclusion since the process of a fracture develops under somewhat different conditions. In fact, an interlaminar fracture takes place in a resin rich interfacial “layer” between the laminate’s plies, although in the intralaminar case it occurs in a matrix–fibre interface. On the other hand, as shown by Garcia et al. [20], modification of a ply interface can affect dynamic behavior of the laminate, especially damping ability, as well as the interlaminar shear strength. The authors compared the characteristics of glass fiber reinforced polymer (GFRP) with and without inclusion of nylon nanofibers among the plies. They found experimentally eigenfrequencies of the composites and determined shear strength in three-point bending. The experimental outcomes were successfully reconstructed numerically in the ANSYS FE software. The authors also studied the influence of polycaprolactone nanofibers on the dynamic and impact properties of GFRPs [21]. Again, they found natural frequencies of laminate beams to be insensitive to structural modifications with nanofibers, but observed stronger damping. In addition, an impact test was performed and simulated with the finite element method. It was shown that separation of the plies with polycaprolactone membranes increased the laminates’ impact resistance and limited the extent of the damage. The relationships between a resistance curve (R-curve), the corresponding fracture process zone length, the shape of the traction-displacement softening law, and the propagation of fracture in the context of the through-the-thickness fracture of composite laminates were examined by Dávila et al. [22]. Oshima et al. [23] have proposed and validated a simple and accurate data reduction scheme for the wedge loaded DCB specimen leading almost to identical values of fracture toughness obtained by the wedge loaded DCB tests and by the standard DCB tests. Czabaj and Ratcliffe [24] compared the intralaminar and interlaminar mode I fracture toughnesses of a unidirectional carbon/epoxy composite and stated that the corresponding fracture energy values were essentially equal. Moreover, they noticed that the observations of the initiation and propagation of intralaminar and interlaminar fracture, and the measurements of fracture toughness, were consistent with fractographic analysis using scanning electron microscopy. Barikani et al. [25] investigated the morphology and the fractography of the Mode I fracture surfaces through thermal analysis using a dynamic mechanical analyzer with the application of the scanning electron microscopy, respectively.

Nowadays, fracture toughness experimental procedures are modified to evade some classical difficulties, such as the precise determination of damage onset or the actual crack length [26,27,28,29,30]. In this trend, the Acoustic Emission (AE) method has gained a growing recognition [31,32,33,34,35]. Of course, there are other good methods for delamination monitoring, such as ultrasonics. For example, Scarponi and Briotti [36] successfully used this technique to assess damage in different composite laminates: the depth and the extension of delamination in the specimens after impact. It is however hard to imagine application of the studied methods in real time, to monitor damage propagation, etc. Other authors [37] tested signal processing techniques applied to ultrasonic waves, towards their ability to resolve echoes generated with delamination processes in carbon fiber reinforced polymer (CFRP) composites. Simulations with two different algorithms were verified experimentally for aircraft laminates. Both algorithms revealed their ability to locate accurately the defects, and, as such, seem to be useful for finding defects in larger structures. Kappatos et al. [38] attempted to evaluate numerically different ultrasonic test configurations for maximal delamination detection effectiveness in CFRPs with various artificial defects. The authors showed that linear array probes working at 5 MHz could provide accurate damage detection in the aircraft composites. Unfortunately, the defect’s shape reconstruction was low and the specimen geometry had to be defined in advance.

On the other hand, the AE technique allows one to supervise both damage initiation and propagation in structures under loads. What is observed and registered are the elastic waves induced by releases of the energy accumulated in a structure in the effect of damage phenomena. The name of the method is rather traditional, as the wave frequencies are usually above 100 kHz and, therefore, cannot be heard. The classical parameters describing the AE signal are the number of counts and the number of hits; both of these parameters competently describe the invisible (internal) defects. However, there is much more information on damage hidden in the raw (full) AE signal, which only recently has been exploited [39] thanks to the significant increase in the speed of computers, as well as the capacities of data storage (the raw data must be sampled at tens of kilohertz, which generates huge files). One of the ample outcomes of the AE research is the frequency analysis in the form of the fast Fourier transform (FFT) applied to the raw AE signal. Keeping in mind that composite materials suffer from several damage forms (matrix cracking, delamination, i.e., interlaminar cleavage, fiber pull-out, fiber breakage) it seems to be a reasonable idea to analyze the FFT spectra in order to recognize both the type and the sequence of damage phenomena occurring in a composite during loading [39]. For example, Saeedifar et al. [40] investigated the evolution behavior of the damage mechanism of quasi-isotropic specimens by the clustering of the AE signals with a hierarchical model. The results of their study show that using the AE technique with an appropriate clustering method, such as hierarchical model, could be an applicable tool for structural health monitoring of composite structures, as well as for detection of barely visible impact damage in laminated composites under quasi-static and dynamic transverse loadings [41].

In this work, the interlaminar fracture of unidirectional composites was studied. The DCB test was used to obtain the fracture energy under mode I loading. The energy release rate values determined using the compliance calibration method, the modified compliance calibration method, and the corrected beam theory were also compared. The main goal was to determine the dominant frequency bands of the released elastic waves related with (i) pure matrix cracking and (ii) the interlaminar cleavage associated with fiber bridging or pullout. The validity of the undertaken research highlights most of the results of Iwamoto et al. [42], who stated that the fiber bridging phenomenon can led to artificially increased value of *G*_IC_ while the intralaminar fracture toughness without bridging fibers had a constant value during cracking.

## 2. Test Specimens

**Figure 1:**
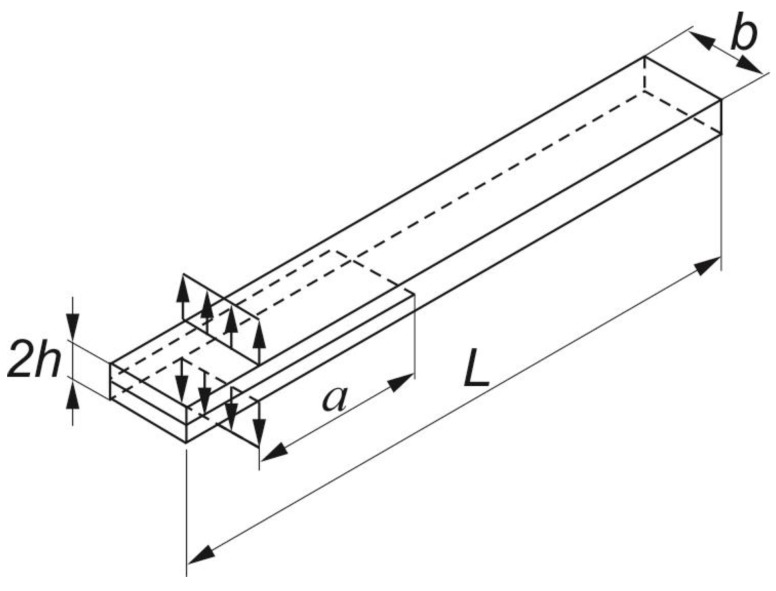
Double cantilever beam (DCB) specimen configuration.

The object of the analysis were specimens predefined to Mode I Interlaminar Fracture Tests of GFRP unidirectional laminates. The specimens taken into consideration had a length of *L* = 125 mm, a width of *b* = 20 mm and a thickness of 2*h*, which equals approximately 4 mm (Figure 1). They were manufactured in accordance with the ASTM D 5528 standard [4]. All the specimens consisted of an even number of plies, namely 16 plies, having 0° orientation (along the longitudinal edges of the sample, cf. Figure 1). Variation in thickness did not exceed 0.1 mm. Between the 8th and the 9th ply, i.e., in the mid-plane, a PTFE (polytetrafluoroethylene) insert was placed to act as the crack starter. The following initial lengths *a* of the pre-cracks were considered: 25 mm and 50 mm. The material selected for the study was SE70/EGL/300g/400mm/35%/PoPa glass-epoxy roving tape with a nominal ply thickness of 0.26 mm.

The specimens were cured with the improved specifications of the autoclaving process parameters, in relation to the recommended settings, with a focus on the manufacturing duration time and the energy consumption (Table 1), but without any significant influence on the obtained material strength characteristics, as well as on the measured elastic properties [43].

## 3. Experimental Procedure

**Figure 2:**
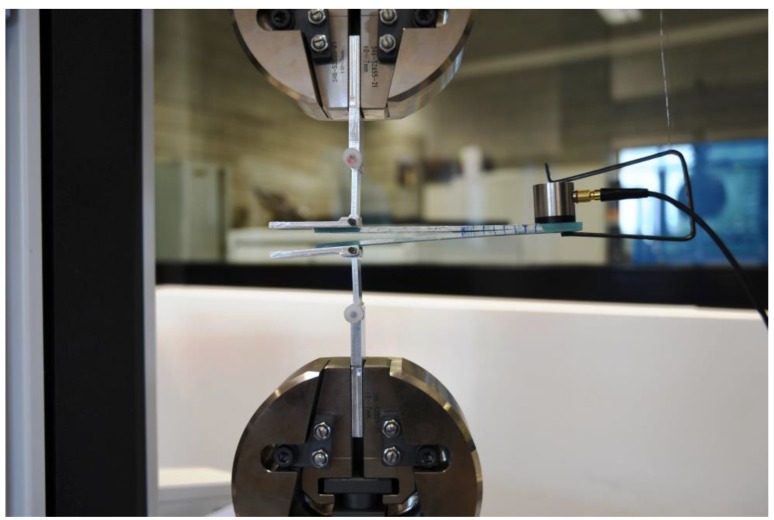
Loaded DCB specimen with AE sensor.

Composite specimens were subjected to the double cantilever (DCB) test in accordance with ASTM D5528 Standard [4]. The experiment was carried out on a Shimadzu ASG-X testing machine with a load cell having a capacity of 1 kN. Figure 2 illustrates the experimental scheme for the DCB testing. The critical value of the strain energy release rate (c-SERR), also denoted as *G*_IC_, i.e., the mode I fracture toughness, of the considered material was determined with three different methods for data reduction: the compliance calibration method (CC), the modified compliance calibration method (MCC), and the corrected beam theory (CBT). The specimens instrumented with piano hinges (own patent No. B1 225617) were mounted in the clamps of the tensile testing machine in such a way that the samples were aligned and centered. The free end of the specimen was slightly supported before loading. Prior to test, each specimen’s edges were coated with typewriter correction fluid and a 50+ millimeter scale was sketched there to enable visual observation of crack propagation. Additionally, a pre-crack cycle was performed. Initiation crack length *a*_0_ was then measured. During the static DCB tests, a displacement control with a constant crosshead rate of 1 mm/min was set to ensure slow and stable delamination growth. Subsequent measurements of the load *P* with the corresponding opening displacement *δ* were conducted. The load-displacement plot was created in real-time with the Trapezium-X software. Delamination onset and all propagation values were visually observed and marked on the specimen edges. Due to difficulties in identification of the initiation point, in the present study two definitions were used: maximum apparent force (*P*_max_) and the one indicated by the acoustic emission (AE). Concerning the former, it was assumed that initiation induced the peak force on the load versus displacement plot. The corresponding point, called *P*_max,_ is not always easy to be identified. Therefore, the latter definition utilizing an acoustic emission technique was applied. The first peak of the AE energy recorded during the test pointed at *P*_max_.

As presented in Figure 2, the AE sensor was attached to each DCB specimen in order to register the elastic waves of AE continuously at a 10 kHz sampling rate. The piezoelectric sensor (“Fujicera” 1045S, Fuji Ceramics Corporation, Yamamiya, Japan, max. 1.3 MHz) was the first element in the Vallen’s “AMSY-5” Acoustic Emission acquisition chain, followed by the “AEP-4” pre-amplifier (34 dB of gain). The next part of the system was the Vallen’s “ASIP-2” analog-to-digital (A/D) card (max. sampling frequency—40 MHz, resolution—18-bit, band width—1.6 to 2400 kHz). In order to synchronize the data from the loading machine and from the Vallen system, the load signal indicated by Shimadzu AGS-X was split and sent to “AMSY-5” in real time. Aside from the counts and the hits, also the amplitude and the energy of the elastic waves were registered, as well as the raw AE signal. The latter was then subjected to the FFT analysis around the onset of delamination, well indicated by the first AE energy peak. This approach reveals the fracture phenomena actually occurring in the structure, with respect to the frequency bands typical for different types of damage [39].

For the mode I DCB tests, the interlaminar fracture toughness in the form of the strain energy release rate (SERR) is expressed as:

(1) $$G_{I}=\frac{P^{2}}{2b}\frac{dC}{da}$$

where *C* is the specimen compliance (the ratio of the load point displacement *δ* to the apparent load *P*); *b*—specimen width and *a*—crack length.

In the experiment, the critical value of SERR (*G*_IC_) was determined by using three different methods. The first one, i.e., the compliance calibration method, is based on the following formula:

(2) $$G_{\mathrm{IC}}=\frac{nP\delta }{2ab}$$

where *n* is a correction parameter which is the slope of *ln(C)* versus *ln(a)* curve. Concerning the modified compliance calibration method, the critical energy release rates can be calculated as

(3) $$G_{\mathrm{IC}}=\frac{3P^{2}C^{2/3}}{2A_{1}bh}$$

where *A*_1_ is the slope of a least squares plot of delamination length normalized by specimen thickness *a/h*, as a function of the cubic root of compliance $C^{1/3}$.

In case of the corrected beam theory:

(4) $$G_{\mathrm{IC}}=\frac{3P\delta }{2b(a+\left|\Delta \right|)}.$$

In Equation (4), Δ is a correction parameter for crack tip rotation and deflection and is determined from linear regression analysis of $C^{1/3}$ versus *a*.

In this paper, calculation of the critical strain energy release rate exploited the values of *P* and *δ* corresponding to delamination initiation, defined for the *P*_max_ and the AE criteria.

## 4. Results and Discussion

The results of the DCB experiments were twofold. The basic outcomes were the initial values of the critical strain energy release rate (fracture toughness) in mode I, *G*_IC_. These values were calculated using three different methods described above. In all of the data reduction schemes determination of the load corresponding to the very onset of delamination was a key point. For this reason, the second type of data was registered in parallel; these data were the AE parameters. Combination of the two sets of data enabled precise determination of *G*_IC_, as well as recognition of damage phenomena taking place in the loaded specimens. The former task was solved with calculation of the numbers given in Table 2. 

The average values of *G*_IC_ obtained from the conducted tests are respectively 0.333, 0.346 and 0.438 N/mm for the CC, CBT and MCC methods, respectively. Although neither the ASTM standard [4] nor round-robin testing [44] suggest the use of any of the methods as being clearly superior to the others, based on the literature review it can be concluded that in most investigations the CBT method yields the most conservative values of *G*_IC_ [23,35]. With regard to the obtained results, the CBT method provides results comparable to those obtained using the CC method. The relative differences in the Mode I interlaminar fracture toughness values determined from both methods were not greater than 9% (criterion: *P*_AE_—8.3%, *P*_max_—8.2%). Significant differences in the values of the critical strain energy release rate were generated by the use of the MCC method. The maximum relative differences of the *G*_IC_ values in relation to the analogous values determined using the CBT or CC method reached almost 45%. This difference is unacceptable and the reason for such discrepancy is now the subject of separate research. As a consequence, in order to determine the final *G*_IC_ value for the material under consideration, the values determined from the MCC method were omitted and the critical strain energy release rate value was finally estimated at 0.34 N/mm. With respect to the obtained results, it should be emphasized that, regardless of the data reduction method used, the relative differences between the results obtained with the implementation of the maximum apparent force criterion (*P*_max_) and those resulting from the application of the AE technique (*P*_AE_) do not exceed 1.6%. This result demonstrates the utility of acoustic emissions and proves that it can be successfully applied to characterize the fracture properties of composites materials.

**Figure 3:**
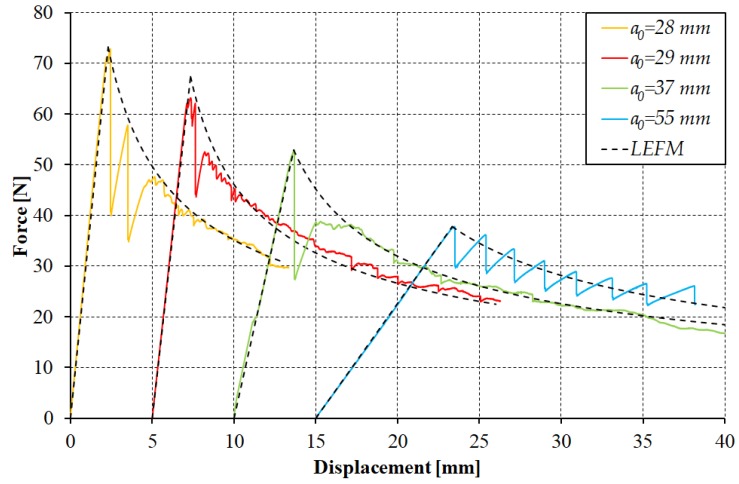
Load-displacement curve obtained from the DCB tests.

In relation to delamination growth, it should be mentioned that generally it may proceed in one of two ways: with a slow and stable propagation or with a run-arrest propagation in which the crack front jumps ahead abruptly. In the case of the analyzed samples, the phenomenon of unstable propagation was an inherent feature of the considered material. The reasons for this behavior are most often seen in the fact that the PTFE insert may not be completely disbonded from the laminate, or may be too thick and cause a large neat resin pocket, or may contain a tear or fold. Despite the fact that only the stable delamination growth is of interest in the ASTM D5528 test method, the experimental studies prove that there are cases in which, despite meeting all the requirements for the inserts used, the material analyzed may still be characterized by an unstable propagation of delamination front [4]. Taking this into account, the considered specimens were unloaded after the first increment of delamination growth and reloaded to continue the test, treating the first delamination growth as the precrack—advised by many. The exemplary load-displacement curves obtained from the DCB test after the first delamination growth are shown in Figure 3.

Assuming the initial length of delamination *a*_0_ was the one after the precrack and the critical strain energy release rate (*G*_IC_) value of 0.34 N/mm, the experimental and analytical curves determined using linear elastic fracture mechanics (LEFM) were compared (Figure 3). It can easily be noticed that the determined *G*_IC_ value allows a reliable reflection of the curves observed in experimental research. The main exception relates to the fact that the analyzed material, during reloading, was still characterized by an unstable propagation of the delamination front, which in turn generated the presence of the peaks and troughs. In most of the analyzed cases, this phenomenon concerned a small range just after reaching the maximum opening force (Figure 3: a_0_ = 28, a_0_ = 29, a_0_ = 37). In some cases, the phenomenon of the unstable delamination growth, leading to a sudden relief of the sample and decrease of the recorded force, was, however, observed in the full range of the test (Figure 3: a_0_ = 55 mm).

The AE parameter chosen to exhibit the onset of the damage was the energy of the elastic wave released as there are some damage phenomena. The energies are usually very small—the basic unit is 10^−18^ J; nevertheless, when damage occurred, the energy peaks were very high (millions of energy units) and as a result, the onset of the damage process could easily be detected. The respective loads were subsequently used to calculate *G*_IC_, as explained above. The results of calculations of the mode I critical strain energy release rate for the specimens with different interfaces obtained by using three different calculations methods are presented in Table 2. 

As mentioned above, the experiments were accompanied by acoustic emission monitoring, which was helpful in precise determination of the peak load respective to the onset of delamination. Nevertheless, registration of the complete AE signal enabled the FFT spectrum analysis. This enabled recognition of different damage forms coming out during the propagation of delamination. The results of the FFT analysis are collected in Table 3. 

The detailed analysis of the FFT spectra revealed three dominating frequency bands of the released elastic waves, characterized with the average values:

- 63.1 kHz—band 1, typical for pure matrix cracking and delamination without fiber bridging or pullout [34],
- 129.5 kHz—band 2, emitted by interlaminar cleavage (delamination) accompanied with the fiber pull-out,
- 213.3 kHz—band 3 connected to fiber breakage.

It should be noted that the intensity (height of the peaks) in the frequency spectra for bands 2 and 3 was much lower (or absent) compared to that of band 1. This can be interpreted as almost pure delamination, as predicted by the ASTM D5528 standard and provided by the DCB experimental setup. On the other hand, the discrepancies in the frequency values observed for band 1 were the biggest (~24% with respect to the average), and may yield from the very nature of the matrix material (epoxy resin), a higher ductility and larger scatter of strength than for the fibers. In any case, the micromechanics of damage processes are a separate area of study due to their complexity, and the differences in the FFT outcomes can normally reach 30%.

**Figure 4:**
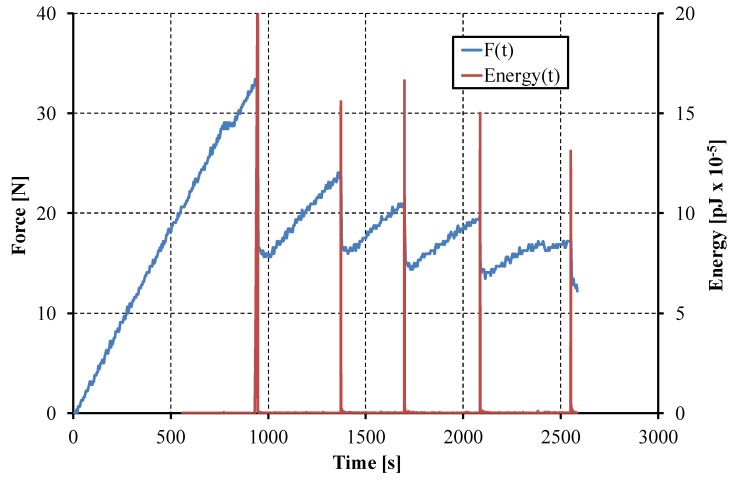
Example of detecting the peak load using the energy of AE.

The exemplary curve of the opening force and the energy of the elastic wave registered during DCB test as a function of time are presented in Figure 4.

## 5. Conclusions

The presented work concerns the analysis of the Mode I interlaminar fracture toughness of the unidirectional GFRP composite material. Determination of the dominant frequency bands of the released elastic (AE) waves related with (i) pure matrix cracking and (ii) the interlaminar cleavage, associated with fiber bridging or pullout, was the main goal undertaken in the first part of the presented study. Analysis of the FFT spectra of the raw acoustic emission signal generated along with the DCB whilst damage (delamination) propagated in the specimens revealed three dominating frequency bands of the released elastic waves. The most intensive wave was in the band of the average frequency equal to ~63 kHz, describing delamination phenomena, which confirmed the assumptions of the DCB test configuration elaborated to provide mode I interlaminar cracking.

The analysis of three methods for experimental data reduction, recommended by the ASTM D5528 standard, seeking to determine the fracture toughness in the form of the critical strain energy release rate (c-SERR or *G*_IC_), was performed. In particular, based on the performed experimental studies, it has been concluded that:

- Mode I interlaminar fracture toughness values determined using the modified compliance calibration method barely correlated with the results obtained with the modified beam theory or with the compliance calibration method,
- Regardless of the data reduction scheme, the differences between the results obtained with the implementation of the maximum apparent force criterion (*P*_max_) and the acoustic emission technique (*P*_AE_) were insignificant,
- The unstable increase of the delamination front was the inherent feature of the material under consideration.

The presented studies revealed also the need for using various methods for data reduction at one time for getting more certainty from the experimental results.

In the second part of the paper, a benchmark study of finite element model validation with the experimental results are presented, where the mesh design techniques are discussed towards improving numerical accuracy and minimizing the computational cost.

## Figures and Tables

**Table 1:** Autoclaving process parameters.

Curing Temperature (°C)	Heating/Cooling Rate (°C/min)	Curing Time (min)	Pressure (MPa)	Vacuum (MPa)
100	1	60	0.4	0.085

**Table 2:** Mode I critical strain energy release rates *G*_IC_ (N/mm).

Initial delamination length *a*_0_ = 25 mm	Sample	Criterion	CC	CBT	MCC
	1	*P*_max_	0.309	0.324	0.480
		*P*_AE_	0.309	0.325	0.482
	2	*P*_max_	0.365	0.370	0.443
		*P*_AE_	0.359	0.364	0.441
	3	*P*_max_	0.309	0.309	0.386
		*P*_AE_	0.307	0.307	0.384
	4	*P*_max_	0.340	0.346	0.356
		*P*_AE_	0.343	0.349	0.360
	5	*P*_max_	0.258	0.279	0.379
		*P*_AE_	0.254	0.275	0.374
Initial delamination length *a*_0_ = 50mm
	6	*P*_max_	0.432	0.436	0.418
		*P*_AE_	0.429	0.433	0.413
	7	*P*_max_	0.335	0.365	0.607
		*P*_AE_	0.333	0.363	0.602

**Table 3:** Results of the fast Fourier transform (FFT) analysis of the raw Acoustic Emission (AE) signals recorded during the DCB tests.

Specimen No.	AE Frequencies (kHz)		
	Band 1	Band 2	Band 3
DCB-2	78.0	115.0	190.0
DCB-3	72.0	125.0	210.0
DCB-4	55.0	‑	‑
DCB-5	72.0	145.0	‑
DCB-6	62.0	120.0	‑
DCB-7	63.0	‑	‑
DCB-8	72.0	142.0	‑
DCB-9	63.0	‑	240.0
DCB-10	44.0	130.0	‑
DCB-11	50.0	‑	‑
Average frequency	63.1	129.5	213.3
maximum	78.0	145.0	240.0
minimum	44.0	115.0	190.0
Relative differences in plus (%)	23.6	12.0	12.5
Relative difference in minus (%)	−24.5	−10.0	−9.7
